# The Controlled Synthesis of Birnessite Nanoflowers *via* H_2_O_2_ Reducing KMnO_4_ For Efficient Adsorption and Photooxidation Activity

**DOI:** 10.3389/fchem.2021.699513

**Published:** 2021-05-26

**Authors:** Yang Li, Guanjie Jiang, Nanqi Ouyang, Zhangjie Qin, Shuai Lan, Qin Zhang

**Affiliations:** Key Laboratory of Poyang Lake Watershed Agricultural Resources and Ecology of Jiangxi Province, College of Land Resources and Environment, Jiangxi Agricultural University, Nanchang, China

**Keywords:** adsorption, birnessite, Pb 2+, phenol, photooxidation

## Abstract

Birnessite nanoflowers composed of layers have been proven to be the strongest adsorbent and oxidant in the surface environment. However, the current synthesis methods of birnessite nanoflowers are suffering from long reaction time and high reaction temperature. Based on these, this paper explores a new method for the rapid and controlled synthesis of layered manganese oxides. The method relies on the molar ratios of KMnO_4_ and H_2_O_2_ redox reacting species to drive the production of birnessite nanoflowers under acidic conditions. The molar ratios of KMnO_4_ and H_2_O_2_ are the key to the crystal structure of the as-prepared. It was found that when the molar ratios of KMnO_4_ and H_2_O_2_ is from 1:1.25 to 1:1.90, the sample is birnessite nanoflowers, and when the ratio is increased to 1:2.0, the sample is a mixture of birnessite nanoflowers and feitknechtite nanoplates. Among the as-prepared samples, BF-1.85 (molar ratios of KMnO_4_ and H_2_O_2_ is 1:1.85) shows the highest capacity for Pb^2+^ adsorption (2,955 mmol/kg) and greatest degradation efficiency of phenol and TOC. The method proposed herein is economical and controllable, and it yields products with high efficiency for the elimination of inorganic and organic pollutants.

## Introduction

The physical and chemical properties of materials are discrepant when they show different morphologies (Li et al., 2020a; Li et al., 2020b). This phenomenon opens up a new perspective for material design (Patzke et al., 2002; Li et al., 2020c). Three-dimensional (3D) hierarchical structures assembly by 1D or 2D nanoscale as building blocks, such as nanosheets, nanoparticles, nanorods, and nanoplates have attracted much attention in recent years (Li et al., 2013; Liao et al., 2013; Wang et al., 2014; Li et al., 2020d; Li et al., 2020e). This is due to the 3D hierarchical architectures not only inherits the superior properties of nanoscale building blocks, but also obtains additional benefits from the unique secondary structure (Yan et al., 2014; Li et al., 2021).

As a common mineral in nature environments, manganese oxide (MnO_2_) is widely used in the fields of energy storage, electrodes, and environmental remediation as catalytic, adsorptive, and oxidative nanomaterial (Jothiramalingam et al., 2006; Singh et al., 2010; Lee et al., 2014; Chen et al., 2016). Hexagonal birnessite is the most common phyllo manganate mineral in the nature environment and consists of flower-like assemblages of ultra-thin 3D nanoplate-shaped crystals (Brousse et al., 2006; Feng et al., 2006; Feng et al., 2007). In fact, most manganese oxides present 3D flower-like layered structure under actual environmental conditions, such as buserite [(Ca_0.5_, Mg_0.5_)O·6MnO_2_·nH_2_O], birnessite and δ-MnO_2_ (Usui and Mita, 1995; Portehault et al., 2008; Nayak et al., 2013; Mayanna et al., 2015; Peng et al., 2015). The primary pathway for the formation of these flower-like manganese oxides is the catalytic oxidation by bacteria (Sasaki and Yu, 2015). Among many flower-shaped manganese oxides, birnessite has been proved to have good photocatalytic activity and heavy metal adsorption capacity (Hou et al., 2017).

At present, birnessite is synthesized via one of three main methods, namely 1) MnO_4_^−^ reduction, 2) Mn^2+^ oxidation, and 3) redox reaction of Mn^2+^ and MnO_4_^−^. In the first method, concentrated HCl, a reducing agent, is reacted with KMnO_4_ to obtain acidic birnessite (Zhao et al., 2009; Su et al., 2019), whereas in the second method, birnessite is generated by passing oxidants, such as O_2_, H_2_O_2_, and Cl_2_, through solutions of MnCl_2_ and NaOH (Yang and Wang, 2001; Feng et al., 2004). Finally, birnessite can also be prepared by the reaction of KMnO_4_ with the Mn(OH)_2_ generated upon mixing MnCl_2_ and NaOH/KOH (Feng et al., 2000). However, these methods are characterized by a long reaction time and require specifically controlled temperatures and/or gas flows, which complicates the reaction conditions. Therefore, there are still many challenges to synthesize birnessite nanoflowers expediently and quickly.

To meet these challenges, Villegas et al. used H_2_O_2_ to reduce KMnO_4_ to obtain crystalline α-MnO_2_ with acidic conditions, for selectively catalytic oxidation of benzyl alcohol and fluorine (Villegas et al., 2005). Compared with the above method, the reduction of KMnO_4_ by H_2_O_2_ under acidic conditions to prepare MnO_2_ not only simplifies the synthetic path and effectively improves the properties of MnO_2_ such as average oxidation state (AOS) and specific surface area (SSA) relating to increase catalytic activity, but also H_2_O_2_ is decomposed into H_2_O and O_2_ during the redox process. Similarly, Chen et al. proposed that the ratio of KMnO_4_ to H_2_O_2_ is the key to controlling the morphology of manganese oxide (Chen et al., 2016). However, the morphology structure of manganese oxide prepared by this method is nanowires, which is not nanoflowers what as expected. Therefore, there are few reports on the preparation of manganese oxide nanoflowers using KMnO_4_ and H_2_O_2_ under acidic conditions.

Herein, a simple method for the synthesis of birnessite nanoflowers is proposed based on the rapid redox reaction between KMnO_4_ and H_2_O_2_ in concentrated H_2_SO_4_ medium. Considering that the amount of acid added is not excessive, and that H_2_O_2_ is decomposed into non-toxic H_2_O and O_2_ in the reaction, the method proposed herein may be characterized as environmentally friendly. In addition, the redox reaction does not require heating and temperature control, neither does it necessitate ageing and recalcination, which ensures high efficiency in short time. To assess the SSA, manganese average oxidation state (Mn_AOS_), Pb^2+^ adsorption capacity, elemental composition, and phenol degradation efficiency of the produced structures, they were analyzed using powder X-ray diffraction (XRD), scanning/transmission electron microscopy (SEM/TEM), and thermogravimetric analysis (TGA). The research results in this paper provide a new perspective to synthesize birnessite nanoflowers by controlling the ratio of reactants, and the synthesized materials possess excellent adsorption and catalytic properties.

## Materials and Methods

### Preparation and Characterization of Manganese Oxide Minerals

**FIGURE 1:**
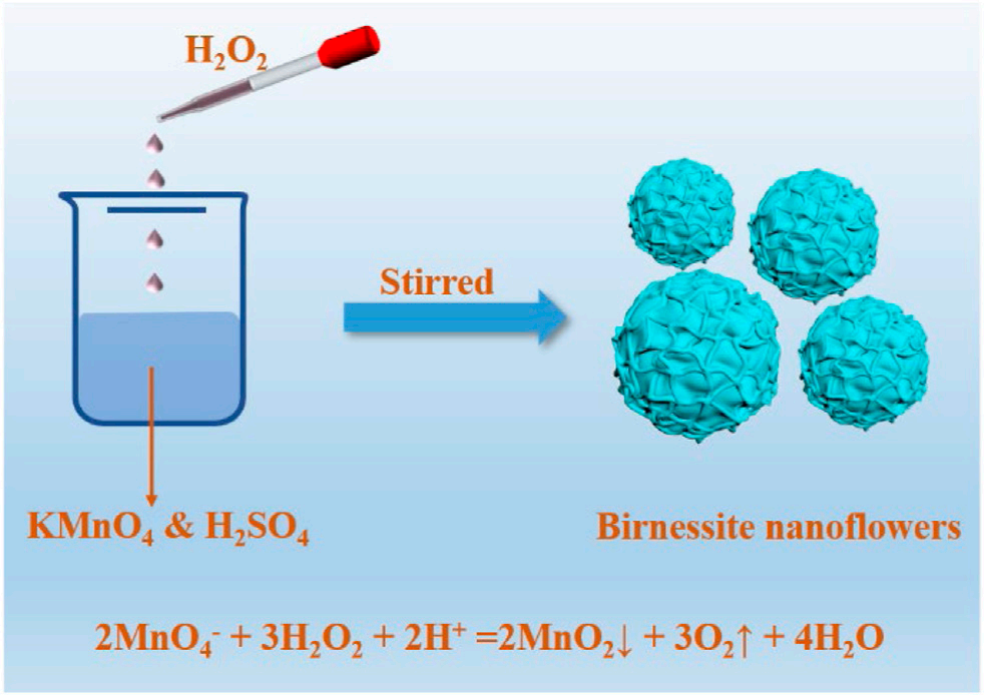
The synthesis process of birnessite nanoflowers by adjusting the ratio of KMnO_4_ to H_2_O_2_.

As showed in Figure 1, the birnessite nanoflowers were prepared by KMnO_4_ and H_2_SO_4_. In detail, 12.7 mmol of KMnO_4_ and 6.33 mmol of concentrated H_2_SO_4_ were added to 100 ml of deionized water. Subsequently, H_2_O_2_ was added and vigorously stirred into the permanganate solution, at the rate of 5 ml/min, using an automatic potentiometric titrator. The amount of hydrogen peroxide added was adjusted so that the KMnO_4_/H_2_O_2_ molar ratios in the reaction mixtures were 1:1.25, 1:1.50, 1:1.75, 1:1.85, 1:1.90, and 1:2.0, leading to BF-1.25, BF-1.50, BF-1.75, BF-1.85, BF-1.90, and BP-2.0 products, respectively. Upon the completion of the reaction, the solid powders were collected and washed with deionized water until a conductivity value less than 20 μS/cm was attained for the filtrate.

The powders were pressed into pellets, then they were analysed by a Brookfield Advance D8 XRD system equipped with a Cu Kα radiation source (λ = 0.15,418 nm). The morphology of the products was observed using an FEI Nova NanoSEM450 cold field emission scanning electron microscope and a JEM-2100F transmission electron microscope. A low-temperature nitrogen adsorption-desorption test was performed using a Micromeritics ASAP 2020 fully automated surface characterization analyser to obtain the SSA of each sample. Meanwhile, thermogravimetric analyses were achieved using a Netzsch STA409PC system. To determine the elemental composition of the products, they were first dissolved in hydroxylamine hydrochloride then analysed by an atomic absorption spectroscope (PerkinElmer AA900) and a flame spectrophotometer. Finally, the manganese oxidation state was assessed according to the oxalic acid method (Kijima et al., 2001).

### Experiments of Pb^2+^ Adsorption on Manganese Oxide Minerals

The efficiency of Pb^2+^ adsorption on the synthesized manganese oxide materials was evaluated using a PerkinElmer AA900 atomic absorption spectrometer. For this purpose, suspensions containing 5 g/L of the mineral sample and 0.1 mol/L of NaNO_3_ were prepared, and their pH values were adjusted to 4.50 using 0.1 mo1/L HNO_3_ and 0.1 mo1/L NaOH. These suspensions were equilibrated for a few days until pH variations less than ± 0.05 within 24 h were established.

In a 50 ml centrifuge tube, 0–8 ml of 15 mmol/L Pb(NO_3_)_2_ solution was diluted to 10 ml with 0.1 mol/L NaNO_3_ and then mixed with 5 ml of the sample suspension, leading to reaction mixtures containing 1.67 g/L manganese oxide mineral and 0–8 mmol/L Pb^2+^. The mixtures were stirred on a magnetic stirrer, and their pH values were adjusted twice to 4.50 ± 0.05 using HNO_3_ and NaOH solution. After 24 h of reaction, the supernatants of the reaction mixtures were filtered through a 0.22 μm membrane then analyzed by atomic absorption spectrometery to determine the concentration of Pb^2+^. The experiments were performed in triplicate for each sample, and the average values are reported herein.

### Experiments of Phenol Degradation by Manganese Oxide Minerals

The effectiveness of the synthesized material in degrading organic pollutant, namely phenol, was determined based on assessments of the total organic carbon (TOC) concentrations, measured using an Elementar Liqui TOC II organic carbon analyzer (Germany). For this purpose, sample suspensions containing 0.625 g/L of the synthesized minerals were prepared and their pH values adjusted to 3.0 using 1 mol/L HCl and 0.1 mo1/L NaOH. Before reacting with phenol, the sample suspensions were equilibrated for a few days until pH variations less than ± 0.05 within 24 h were established. Subsequently, they were added to phenol in a 250 ml double-layer beaker, and allowed to react for 6 h. The temperature of the mixtures initially containing 0.5 g/L manganese oxide mineral and 100 ppm phenol was maintained at 25°C throughout the reaction using a low-temperature circulating cooling water system. Upon the termination of the reaction, the solution was filtered through a 0.22 μm membrane, and the supernatant was analysed by an Agilent high performance liquid chromatography system (ultraviolet (UV) detector, 70% water and 30% methanol mobile phase, flow rate = 0.3 ml/min, column temperature = 30°C) to determine the residual phenol concentration. The total organic carbon (TOC) concentration in the supernatant was also assessed using an Elementar Liqui TOC II organic carbon analyzer (Germany). The analyses are performed in duplicate, and only the average values are reported herein.

## Results and Discussion

### Characterization of the Synthesized Manganese Oxide Minerals

#### XRD and FTIR Analysis

**FIGURE 2:**
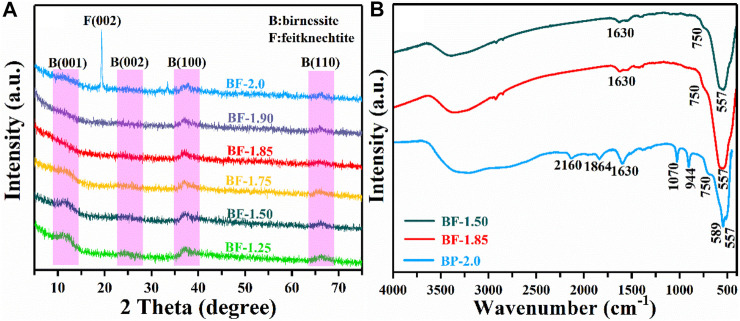
**(A)** XRD and **(B)** FTIR patterns of as-prepared samples.

Figure 2A shows the XRD patterns of the as-prepared samples by the reaction of KMnO_4_ with H_2_O_2_ at different molar ratios. When the molar ratio of KMnO_4_ and H_2_O_2_ are 1:1.25, 1:1.50, and 1:1.75, the powder XRD pattern (Figure 2A) appeared four characteristic diffraction peaks (001), (002), (100), and (110), respectively, which are basically consistent with the acid birnessite (JCPDS: 80-1098). Surprisingly, a characteristic peak is appeared at 33.4°, which may correspond to vernadite. This is because when the Mn_AOS_ is low, vernadite and feitknechtite are easy to transform (Grangeon et al., 2017). Upon increasing the KMnO_4_/H_2_O_2_ molar ratio to 1:2, the manganese oxide products show the (002) and (006) planes of feitknechtite (JCPDS: 18-804), as well as the (100) and (110) plane of birnessite. These results indicate that the materials produced with the ratio to 1:2 KMnO_4_/H_2_O_2_ reaction mixtures are composed of both feitknechtite and birnessite. To accurately explore the molar ratio of KMnO_4_ and H_2_O_2_ in the phase transition of birnessite, BF-1.85 and BF-1.90 samples were further prepared by adjusting the proportion of KMnO_4_ and H_2_O_2_ to 1:1.85 and 1:1.90. As shown in Figure 2A, the XRD patterns of BF-1.85 and BF-1.90 samples only show the (100) and (110) plane of birnessite. The disappearance of the (001) and (002) diffraction peaks is considered to be an important distinctive feature between feitknechtite and birnessite (Post, 1999; Villalobos et al., 2003). These results prove the phase transition of birnessite begins with KMnO_4_/H_2_O_2_ of 1:2. The results confirm that birnessite is successfully synthesized simply by changing the molar ratio of KMnO_4_ to H_2_O_2_ in the reaction, and the concentration of H_2_O_2_ plays a key role in the crystal phase of the product. Based on the high intensities of their diffraction peaks, BF-1.50, BF-1.85, and BP-2.0 were selected as representative samples for further study. The characterizations of BF-1.25, BF-1.75, and BF-1.95 were still detected and presented in Supplementary Figures S1–S4.

The structure of the as-prepared samples was further confirmed by FTIR patterns. As shown in Figure 2B, BF-1.50 and BF-1.85 present the same characteristic peak, corresponding to the birnessite (Zhang et al., 2018). Similarly, the characteristic peak of BF-1.25, BF-1.75, and BF-1.95 are derived from the structure of birnessite (Supplementary Figure S1). When the molar ratio of KMnO_4_ and H_2_O_2_ increases to 1:2.0, the new characteristic peak of BP-2.0 appears obviously at 944 and 1,070 cm^−1^. It indicates the structure of BP-2.0 is mixture of feitknechtite and birnessite (Sharma and Whittingham, 2001; Zhao et al., 2012), which is consistent with the results of XRD.

#### Scanning Electron Microscopy and High-Resolution Transmission Electron Microscopy

**FIGURE 3:**
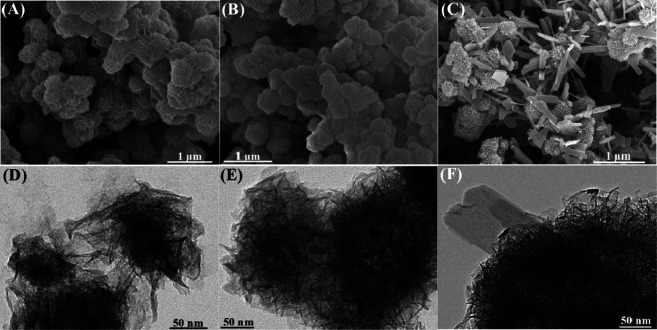
SEM and HRTEM images of **(A,D)** BF-1.50, **(B,E)** BF-1.85 and **(C,F)** BP-2.0.

Field emission scanning electron microscopy (SEM) was performed to observe the morphologies of as prepared samples. As shown in Figure 3, BF-1.50 and BF-1.85 samples have a uniform morphology and a three-dimensional spherical flower-like shape with diameters of about 0.5–1 and 0.3–0.5 μm, respectively, (Figures 3A,B). In detail, the particle size gradually decreases with the increasing amount of hydrogen peroxide. Besides, the mineral morphology changes from nano-flower spheres to rough-surface spheres. Matching with XRD results, the morphology of BP-2.0 is the mixture of nanoflowers and nanoplates (Figure 3C) with phase transition. Thereinto, the nanoflowers, 0.3–0.5 μm in diameter, gradually aggregate and transform into nanoplates, and the width and length of nanoplates are about 60–200 and 300–800 nm, respectively. Moreover, the images recorded by HRTEM are similar to those captured by SEM. Figures 3D,E further confirm that the morphology of BF-1.50 and BF-1.85 are spherical composed of two-dimensional sheets, while BP-2.0 is the mixture of nanoflowers and nanoplates. These results show that beyond a specific value, H_2_O_2_ content in the reaction mixture has a considerable influence on the mineral morphology and particle size of the synthesized material.

#### TGA

**FIGURE 4:**
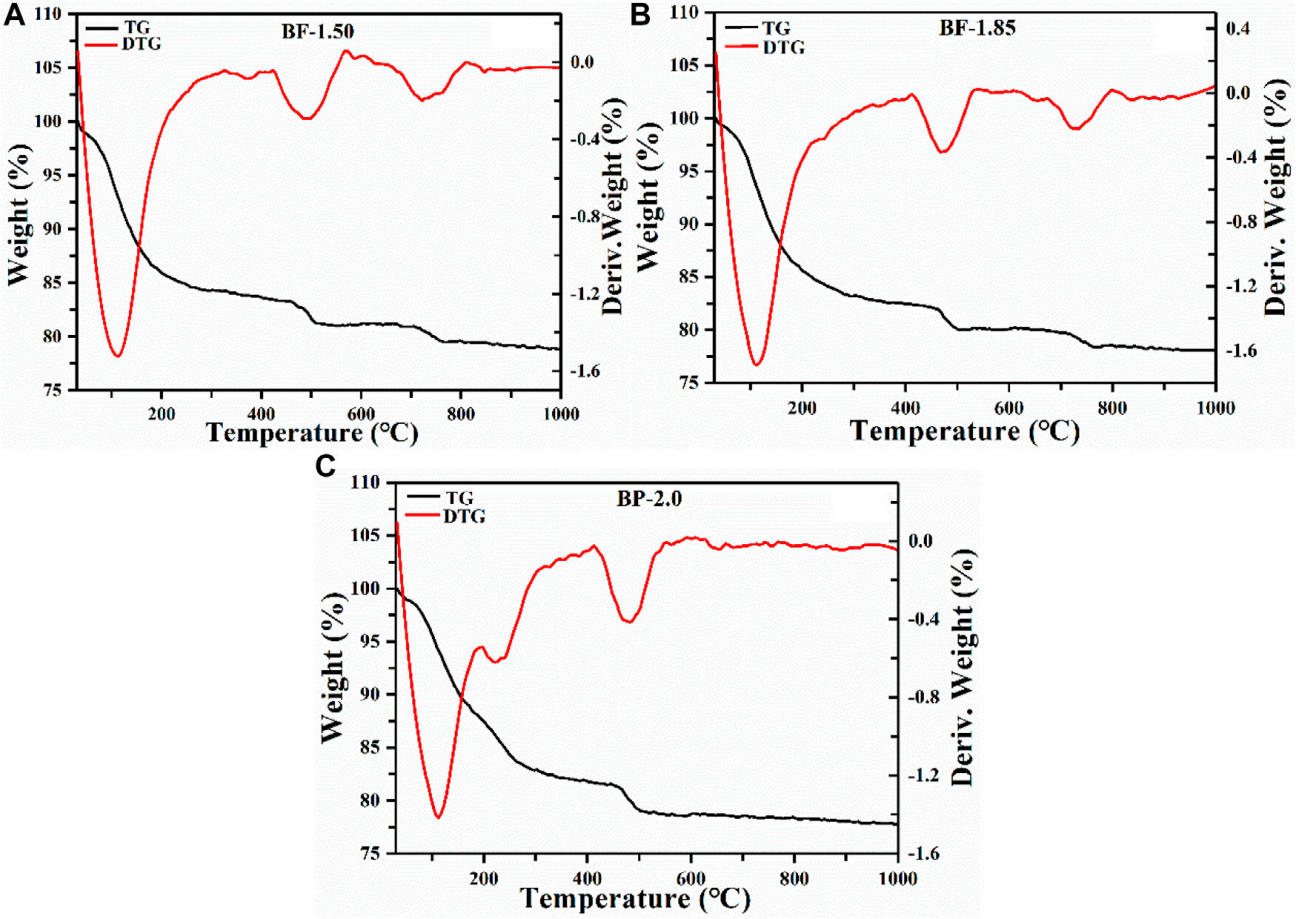
TG and DTG curves of **(A)** BF-1.50, **(B)** BF-1.85 and **(C)** BP-2.0.

The thermogravimetric curves presented in Figure 4 show that all manganese oxide samples exhibit similar total weight losses of about 20.1–21.9%. The weight is lost due to 1) the desorption of physically adsorbed water on the mineral surface (Cheney et al., 2008) and 2) the transfer of chemically bound water molecules between mineral layers (Yang and Wang, 2001), as well as the 3) reaction of some O atoms and Mn^4+^ ions in the manganese-oxygen octahedral layers to form single Mn_2_O_3_ products (Chen et al., 1996). In BP-2.0, weight loss processes (1), (2), and (3) occur at temperatures of 0–192, 192–412, and 412–610°C, respectively. The loss rate of bound water in the second process is fast, and the amount of weight lost is pronounced at around 490°C during the third process wherein the release of lattice oxygen might lead to a certain degree of lattice collapse. Meanwhile, in BF and BF-1.85 samples, weight loss are mainly due to the transfer of water molecules between layers. Beyond 610°C, losses in these materials become negligible, indicating they may have been converted to Mn_3_O_4_ and Mn_2_O_3_, so that there is no more oxygen being released (Qin et al., 2016). Compared to BF- and BF-1.85, the BP-2.0 exhibits weight loss at lower temperatures during all three processes, suggesting that it is less thermally stable. These results indicate that the enhancement in the molar ratio of KMnO_4_ and H_2_O_2_ will reduce the thermal stability of the product.

#### Manganese Oxidation State, SSA, and Chemical Composition

Table 1 summarizes the oxidation states and chemical compositions of the MnO_2_ mineral samples synthesized using different molar ratios of KMnO_4_/H_2_O_2_. For all products, the AOS of manganese is within 3.71–3.87, indicating that Mn(IV) is the dominant state of the element, with a small amount of low-oxidation-state manganese being present. Furthermore, the summarized values suggest that Mn_AOS_ and K content can decrease with increasing H_2_O_2_ fractions; however, variations in Mn content are unsubstantial. The decrease in K content is actually quite dramatic. It probably because the increasing H_2_O_2_ fractions in reducing oxidation state, diminishing the number of octahedral vacancies, and decreasing the number of negative charges, which lead to reduce K^+^ charges between the equilibrium layers (Sakai et al., 2005; Hsu et al., 2012; Liu et al., 2016). SSA indirectly reflect the size and bulk density of aggregated crystals. The SSA value of BF-1.50 and BF-1.85 are 103 and 173 m^2^/g, respectively. Considering that the BP-2.0 sample contains a significant amount of feitknechtite, its SSA value of 185 m^2^/g is also relatively large.

**TABLE 1:** Chemical components, AOS and SSA of manganese oxide samples prepared using reaction mixtures of varying KMnO_4_/H_2_O_2_ ratios.

Sample	Mn_AOS_	SSA (m^2^ g^−1^)	Elemental content (%)		Chemical components
			Mn	K	
BF-1.50	3.87	103	46.49	2.44	K_0.07_MnO_1.97_(H_2_O)_0.60_
BF-1.85	3.71	173	47.95	1.05	K_0.03_MnO_1.87_(H_2_O)_0.78_
BP-2.0	3.75	185	44.73	0.85	K_0.03_MnO_1.89_(H_2_O)_0.76_

### Adsorption of Pb^2+^

**FIGURE 5:**
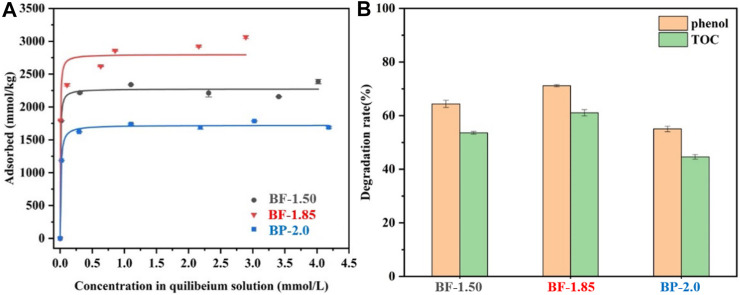
**(A)** Pb^2+^ uptake isotherms, **(B)** Elimination of TOC and phenol of BF-1.50, BF-1.85, and BP-2.0.

The isothermal L-shaped adsorption fitting curves of Pb^2+^ on BF-x and BP-2.0 are shown in Figure 5A. These curves indicate that BF-1.85 has the greatest capacity for Pb^2+^ adsorption, followed by BF-1.50 and then BP-2.0. The Langmuir fitted maximum adsorption capacity values of all samples are listed in Table 2, and as expected, they are strongly correlated with other related parameters, such as SSA and Mn_AOS_. With a SSA of 173 m^2^/g and a maximum capacity of 2,955 mmol/kg, the BF-1.85 sample exhibit excellent Pb^2+^ adsorption performance. In the samples, lead is mainly present in the form of a triple-corner-sharing (TCS) or a triple-edge-sharing (TES) inner-sphere complex above or below the octahedral vacancy. Otherwise, it is adsorbed at double-corner-sharing (DCS) or double-edge-sharing (DES) edge surface sites (Burn-Nunes et al., 2011; Wang et al., 2012). The SSA of BF-1.85 is large, and its (001) crystal plane is rarely stacked; therefore, it has numerous exposed active sites available for the adsorption of Pb^2+^. By calculation, the lead adsorption densities of BF-1.50, BF-1.85, and BP-2.0 are 22.1, 17.1, and 9.3 μmol/m^2^, respectively. The difference in adsorption capacity is mainly attributed to the variation in SSA (the latter has a much larger surface area). Even though BP-2.0 has a relatively large SSA, the nanoplates structure in its morphology have few active sites, which can lead to a small Pb^2+^ adsorption capacity.

**TABLE 2:** Langmuir parameters of Pb^2+^ adsorption on BF-1.50, BF-1.85, and BP-2.0.

Sample	A_max_(mmol/kg)	K	*R* ^2^
BF-1.50	2,274	250	0.99
BF-1.85	2,955	33.3	0.988
BP-2.0	1724	111	0.996

### Degradation of Phenol

The efficiencies of BF-1.50, BF-1.85, and BP-2.0 in eliminating phenol organic pollutants were assessed based on residual phenol and total organic carbon (TOC) measurements (Figure 5B). The order of phenol and TOC removal rate is as follows: BF-1.85 > BF-1.50 > BP-2.0. All three samples exhibit distinguished elimination activity, and BF-1.85 shows the best performance in degradation of phenol. The process of phenol degradation on manganese oxide is that phenol is adsorbed onto its surface, followed by subsequent oxidation (Hu et al., 2010). The large SSA of manganese oxides with more active sites is conducive to the adsorption and later oxidation of phenol (Chen et al., 2018). Therefore, BF-1.85 is a nanomaterial with highest efficiency of degradation capacity in three samples because of its large SSA. The degradation rate of BF-1.50 is only lower than Ver-1.85. In general, the higher the Mn_AOS_ for manganese oxides, the more favourable will be the oxidation reaction (Nayak et al., 2013). However, as the SSA of BF-1.50 is nearly 40% smaller than Ver-1.85, there are fewer surface-active sites. It can be explained that the decrease of SSA has a greater effect on degradation of phenol than that of high Mn_AOS_, so the elimination ability to phenol of BF-1.50 is lower than BF-1.85. Conversely, BP-2.0 has a similar Mn_AOS_ and SSA to BF-1.85, but the degradation rate of phenol and TOC is 22.6 and 46.9% lower than those of BF-1.85. The result can be related to mixed mineral phase of this sample. Since a large amount of birnessite in BP-2.0 is converted into feitknechtite, its elimination efficiency for phenol and TOC is reduced.

## Conclusion

This study investigates the effect of KMnO_4_/H_2_O_2_ ratios on the physicochemical properties, structure, adsorption capacity, and degradation efficiency of manganese oxide products prepared by the simple method of reacting permanganate solutions with hydrogen peroxide. Birnessite and a mixture of feitknechtite and birnessite products are generated sequentially upon increasing the ratio of KMnO_4_ to H_2_O_2_. In general, the synthesized nanomaterials are characterized by large SSA and high manganese oxidation state, and they show good performances in terms of Pb^2+^ adsorption and phenol removal. Although the structures and properties are not uncommon, they are well related to the synthetic procedure. Manganese oxide has attracted much attention as a material that can remove inorganic and organic pollutants, therefore, it is of great significance to obtain different pure phase manganese oxides only by controlling the proportion of reactants, and it also has great potential for using on an industrial scale. Moreover, it also provides a reference for the development of other effective and environmentally friendly methods to be used in the preparation of novel materials.

## Data Availability

The original contributions presented in the study are included in the article/Supplementary Material, further inquiries can be directed to the corresponding author.
