# Supplementary material for: The Controlled Synthesis of Birnessite Nanoflowers via H2O2 Reducing KMnO4 For Efficient Adsorption and Photooxidation Activity
Source: Front Chem. 2021 May 26;9:699513. doi: 10.3389/fchem.2021.699513 (PMC8187863; doi:10.3389/fchem.2021.699513)
Supplement: Supplementary file 1 [file DataSheet1.docx]

The Controlled Synthesis of Birnessite Nanoflowers *via* H_2_O_2_ Reducing KMnO_4_ for Efficient Adsorption and Photooxidation Activity

Yang Li^1^, Guangjie Jiang^1^, Nanqi Ouyang, Zhangjie Qin, Shuai Lan, and Qin Zhang*

Key Laboratory of Poyang Lake Basin Agricultural Resource and Ecology of Jiangxi Province, College of Land Resource and Environment, Jiangxi Agricultural University, Nanchang 330045, China, E-mail: chincheung@live.com





**Figure S1** FTIR patterns of BF-1.25, BF-1.75 and BF-1.90.


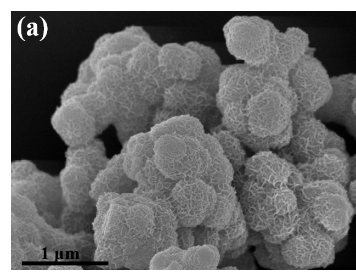

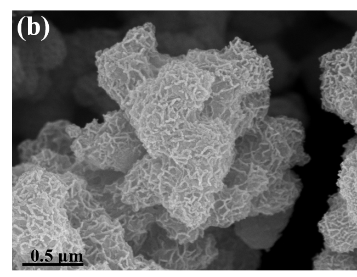

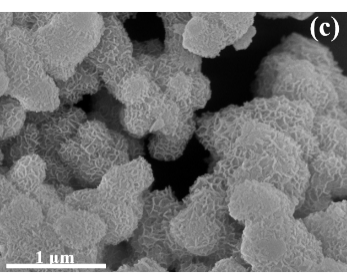


**Figure S2** SEM images of (a) BF-1.25, (b) BF-1.75 and (c) BF-1.95.


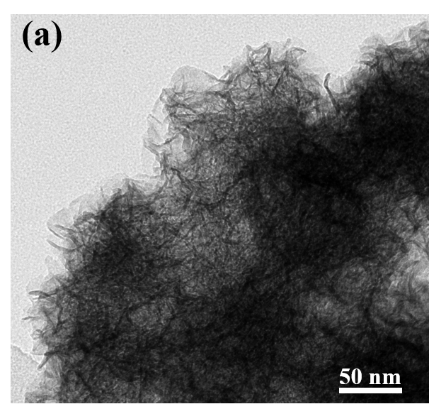

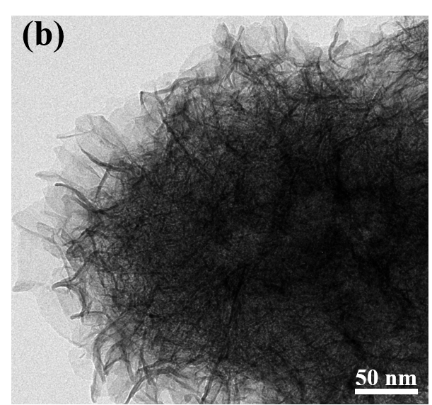

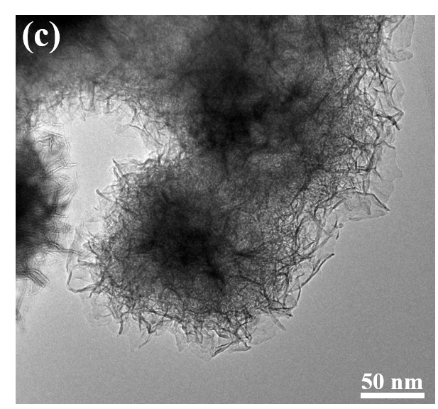


**Figure S3** HRTEM images of (a) BF-1.25, (b) BF-1.75 and (c) BF-1.95.










**Figure S4** TG and DTG curves of (a) BF-1.25, (b) BF-1.75 and (c) BF-1.90.
